# Stressor Combat Strategies and Motivating Factors Among Health Care Service Providers During COVID-19 Pandemic

**DOI:** 10.7759/cureus.14726

**Published:** 2021-04-27

**Authors:** Anurag Srivastava, Saurabh Srivastava, Rashmi Upadhyay, Rakesh Gupta, Kiran Jakhar, Ruchi Pandey

**Affiliations:** 1 Community Medicine, Government Institute of Medical Sciences, Greater Noida, IND; 2 Internal Medicine, Government Institute of Medical Sciences, Greater Noida, IND; 3 Pulmonary Medicine, Government Institute of Medical Sciences, Greater Noida, IND; 4 Psychiatry, Government Institute of Medical Sciences, Greater Noida, IND

**Keywords:** covid 19, stressor, pandemic, health care workers, severe acute respiratory syndrome coronavirus-2 (sars-cov-2)

## Abstract

Context

Since its inception in December 2019 in Wuhan, China, the severe acute respiratory syndrome coronavirus-2, the etiological agent for coronavirus disease 2019 (COVID-19), is spreading rapidly both locally and internationally, and became certified as a pandemic by the World Health Organization (WHO) in March 2020. Working in an environment of high risk, coupled with adherence to quarantine and stressors related to the job, has been found to exacerbate the psychological health of frontline healthcare workers.

Aims

To assess the perceived stressors, combat strategies, and motivating factors among health care service providers during the COVID-19 pandemic.

Setting and design

A cross-sectional study was conducted among healthcare workers at a tertiary care hospital in the north-central region of India from May to September 2020.

Methods and materials

A convenience sample of 150 health care workers was taken. A self-reported pretested structured “COVID 19 staff questionnaire” was used as a study tool. The health care workers (HCWs) included nurses, physicians, laboratory technicians, and radiology technicians who worked in high-risk areas (isolation ward, COVID intensive care unit, emergency department, and outpatient cough outdoor walk-in clinics) during the outbreak constituted our study population.

Statistical analysis used

The varying levels of stress or effectiveness of measures were reported as mean and standard deviation, as appropriate. Descriptive statistics were used for data presentation. A Mann-Whitney U test was used to analyse differences between two groups of non-normally distributed data. A p-value of less than 0.05 was considered statistically significant.

Results

As compared to doctors, paramedical staffs were more stressed with frequent protocol changes (88%), emotional exhaustion (68%), and conflicts with duties (62.7%). The factors like seeing colleague getting better (78.7%) and hoping for financial compensation (49.3%) were reported as stress busters; family compensation in case of death at the workplace and disability benefits in case of disease-related disability development were more effective motivational factors for paramedical staff in case of future outbreaks (p-value <0.05).

Conclusions

It is needful that secondarily traumatized team members should be always observed, educated, and properly handled. Certain personal coping strategies adopted by health workers should be well addressed and motivated if scientifically sustainable. We have to include psychiatric preparedness and stress monitoring also for health care teams along with emphasizing hygiene, temperature monitoring, and fever management, in planning to fight the pandemic.

## Introduction

Infectious diseases have beleaguered all sectors of society since antiquity such as education, economy, international relations, and most importantly, its healthcare system. Of the very first responses of the country, the healthcare system ubiquitously swings into action to contain the spread and transmission of the disease.

Since its inception in December 2019 in Wuhan in China, the severe acute respiratory syndrome coronavirus-2 (SARS-CoV-2), the etiological agent for coronavirus disease 2019 (COVID-19), is spreading rapidly both locally and internationally [1]. The virus-caused disease was declared a public health emergency by the World Health Organization (WHO) in less than a month and was declared a pandemic by WHO in March 2020. Despite its low mortality rate of 2%, SARS-CoV-2 is extremely infectious, and the mortality rate is higher than that of previous pandemics such as severe acute respiratory syndrome (SARS) and Middle East respiratory syndrome (MERS) [2].

While this disease developed in 213 countries throughout the world, health care workers were involved in the screening and treatment, and as result of which they were are at risk of exposure to the highly infectious virus during patient care or by exposure to patient environment or through biological samples. Frontline healthcare workers are always at a high state of vulnerability to the severe strain of being infected and transmitting the infection to their family members. Working in a high-risk environment, coupled with adherence to quarantine and stressors related to the job, has been found to exacerbate their psychological health [3]. The main psychological concerns were regarding the safety of self, colleagues, and family members. Seeing a colleague getting sick after contracting an infection, caring for sick colleagues, and getting intubated for treatment were high-stress variables selected by Health Care Workers (HCWs) [4]. No one knows how long the uncertainty is going to last, and that has had an exaggerated impact on the psychological health of healthcare workers. An earlier study on the MERS outbreak has shown that a positive attitude at the workplace has a paramount place in reducing stress among HCWs. Additionally, conditions like infected staffs getting better, adequate availability of protective equipment, and eventual drop in disease prevalence with strict infection control measures have shown to reduce stress and anxiety among HCWs [4].

Added to the aforementioned psychosocial ramifications, there is also colossal literature on how these factors can have more persistent effects on the physical and cognitive abilities of these HCWs, thereby jeopardizing their workplace performance. Such mental health issues can have deleterious repercussions on their attention, decision-making abilities, as well as their overall wellbeing [5]. Evidence accumulated from the epicentre of the outbreak (Wuhan, China) has also proved that the catastrophe has led to deleterious psychosocial ramifications on HCWs, some of which include anxiety, depressive symptoms, fear, and anger [6].

Previous research has identified core messages that healthcare professionals have requested from their employer during the COVID-19 pandemic which are: “hear me, protect me, prepare me, support me, and care for me” [7]. In order to perform the responsibilities to their fullest potential over an unpredicted time period, the healthcare authorities must have provisions for early essential psychosocial support for all HCWs that properly respond to these requests and are focused on the establishment of a nontoxic psychological environment. Given the biopsychosocial impact of working in high-risk environments during the COVID-19 pandemic, it becomes imperative that researchers investigate the potential influencing factors, stress-combating, and motivational factors employed to ensure that the HCWs are able to perform their duties optimally. Hence there is an exigent need to assess the psychological health of HCWs at COVID hospitals in the current scenario of the COVID-19 pandemic in India. That would also be helpful to apprise the relevant healthcare authorities regarding necessary steps to be taken to ensure the psychosocial safety of our frontline warriors.

Recognizing this need, the study was planned with the aim to assess stressor combat strategies and motivating factors among health care service providers during the COVID-19 pandemic.

## Materials and methods

This study was conducted in accordance with the Declaration of Helsinki [8]. All participants registered their consent before participation. Ethical permission for the study was obtained from the Institute’s Ethical Committee (REF# GIMS/IEC/HR/EFR/2020/11).

Study site

The current study was conducted among healthcare workers working in a tertiary care hospital designated L2 COVID-19 care centre in the north-central region of India in the state of Uttar Pradesh. The hospital had a total of 150 beds, including 140 beds in the isolation ward and 10 medical intensive care unit (ICU) beds. During the COVID-19 outbreak, a total of 400 confirmed cases of COVID-19 were admitted at our hospital from March to June 2020, including 23 hospital staff. All the admitted hospital staff recovered from COVID-19. 

Study design

This observational and cross-sectional study was conducted from 1 May to 31 August 2020, which was the peak emerging period of COVID-19 outbreak in India. 

Study subjects and sample size 

The health care workers (HCWs), including nurses, physicians, laboratory technicians, and radiology technicians who worked in high-risk areas (isolation ward, COVID ICU, emergency department, and flu out patient department [OPD]) during the outbreak were the study universe. For the purpose of the study, the jobs of hospital workers were classified into two categories: (1) doctors and (2) paramedical staff, including clinical technical/support staff (nursing staff, radiological technologists, clinical, and laboratory technicians). The healthcare workers estimated to be at risk were 250, among whom a convenience sample of 150 HCWs was taken for the study. Out of 150 HCWs, 48 were doctors and 102 were paramedical staff.

Study tool 

We developed and administered a self-reported “COVID-19 staff questionnaire” to study participants, consisting of two parts. This questionnaire was modified from the “MERS CoV staff questionnaire" [4], which was originally adapted from Lee et al. [9]. Part A of the questionnaire consisted of sociodemographic data of the study participants, in­cluding age, sex, educational, and marital status. Professional and work-related information constituted their title, income, professional experience, and area of work. Part B of the questionnaire was derived and modified from the one used by Lee et al. among the hospital staff during the SARS epidemic in 2003 [9] and Khalid et al. during MERS CoV epidemic [4]. Part B of the questionnaire originally consisted of 5 sections with 82 questions in English. These sections captured the emotions, perceived stressors, factors that reduced their stress, coping strategies, and motivators to work during future missions of the health care workers. The responses scoring was on a scale from 0-3. The responses were reported as mean and standard deviation.

The questionnaire was pilot tested with 10 HCWs. After obtaining the feedback and response, thelanguage of Part B was modified and streamlined. The final version of part B consisted of 5 sections with 72 questions in English .

The first section of the questionnaire consisted of 15 questions that captured the emotions of the staff during the COVID-19 outbreak. Each question had four choices based on the severity of the feelings on a 4-point scale (0=not at all; 1=slight; 2=moderate; 3=very much). The internal consistency coefficients were 0.71 (Kuder-Richardson Formula 20) for the score on their feelings and 0.78 (Cronbach’s α) for the severity of feelings.

The second section evaluated 20 different possible factors that could have caused stress among the staff. Responses were taken on a 4-point scale based on the severity of the stress factor (0=very minimal; 1=slight; 2=moderate; 3=very much). The internal consistency coefficients were 0.83 (Kuder-Richardson Formula 20) for the number of stressors and 0.89 (Cronbach’s α) for stress severity. 

The third section consisted of had 14 questions assessing different factors, the availability of which helped reduce the stress of HCWs, either directly or indirectly. The reply was again on a 4-point scale (0=not at all effective; 1=mildly effective; 2=moderately effective; 3=extremely effective). The internal consistency coefficient (Cronbach’s α) for the degree of effectiveness was 0.84.

Similarly, the section four with 13 questions assessed different personal coping strategies of HCWs that were rated on 4-point scale from 0-3 (0=never; 1=sometimes; 2=often; 3=always). The internal consistency coefficients were 0.74 (Kuder-Richardson Formula 20) for the number of stressors and 0.79 (Cronbach’s α) for the rating of coping strategies.

Additionally, the fifth section had 10 questions that explored the possibility of the willingness of HCWs to participate in any future COVID-19 or other epidemics. These were rated on a 4-point scale (0=not at all important to 4=most important) [4].

The final version was administered to a convenience sample of 150 HCWs who participated voluntarily in the study. All targeted staff worked in the high-risk areas of the hospital where they were continuously at risk of exposure to COVID-19 patients. The staff registered their responses electronically on Google forms (Google LLC, Menlo Park, CA, USA). The study questionnaire was administered to HCWs during their stay in passive quarantine. All subjects gave informed written consent before completing the online form.

Prior to conducting the survey, the participants were informed about the purpose of the study and how it was going to be conducted. All had submitted their informed consent prior to the response. The participants had the right to withdraw from the study at any time without any negative impact on them. The personal information received was also not disclosed to anyone except the researcher. The link of the study tool was sent electronically to respondents in order to maintain their privacy and to ensure the prevention of any psychological discomfort when the respondents were not able to respond. 

Data analysis and statistical analysis

Descriptive statistics were used to present the preliminary findings of the study. Categorical variables were presented by percentage. The different levels of stress and coping were represented as mean and standard deviation. A Mann-Whitney U test was used to analyse differences between two groups of nonnormally distributed data. A p-value of less than 0.05 was considered statistically significant.

## Results

Out of total 163 eligible staff, 150 (92%) responded. 2 duplicate responses were excluded. The mean age of participants was 32.3 years for doctors and 39.7 years for paramedics, among doctors sex ratio was almost equal but in paramedics’ females predominated in distribution. More than 80% of the paramedics were married and about 60% of medicos were married. The mean duration of professional experience of doctors was 6.8 years and 7.3 years for the paramedics (Table 1).

**Table 1:** Biosocial characteristics of health care staff

Characteristics	Doctors (N=48)			Paramedical Staff (N=102)		
	n	%	Mean (SD)	n	%	Mean (SD)
Age-wise distribution						
20-30 years	25	52.1	32.3 (2.3) years	33	32.3	39.7 (5.2) years
31-40 years	16	33.3		43	42.2	
41-50 years	7	14.6		26	25.5	
Place of work/professional experience						
≤ 1 year	4	8.4	6.8 (4.7) years	8	7.8	7.3 (3.2) years
1-5 years	13	27.1		40	39.3	
> 5 years	31	64.6		54	52.9	
Sex						
Male	26	54.2		23	22.5	
Female	22	45.8		79	77.5	
Marital Status						
Married	28	58.3		84	82.4	
Unmarried	20	41.7		18	17.6	

The most commonly perceived feeling, to which whole the staff responded positively, was the innate professional and ethical commitment along with special recognition by hospital administration that boosted the staff to continue their work during the pandemic. However, the staff also felt distressed during the outbreak but appreciated the extra financial compensation after the outbreak. They even tried to limit their exposure to patients with COVID-19, and were particularly unhappy to work overtime. In case a COVID-19 outbreak recurred, only a few health workers (30.7%) were thinking of quitting their jobs. Paramedical staff in the study felt more nervous, tried quitting their jobs, expected salary hike, and shortened their interaction with the patients as compared to their medical counterparts, the difference of which was also statistically significant (p<0.05) (Table 2).

**Table 2:** Feelings of HCWs who were directly involved in taking care of patients during COVID-19 outbreak * Based on Mann-Whitney U test ^s ^Statistically significant HCW: healthcare workers; SD: standard deviation

No.	Feelings of HCWs during COVID-19 outbreak	Positive Answer		Mean (SD)		p-value*
		n	%	Doctors (N=48)	Paramedical (N=102)	
1	You felt that you had to do your job as it was your professional and ethical duty	150	100	2.83 (0.84)	2.18 (0.78)	0.189
2	You felt nervous and scared	65	43.3	1.38 (0.64)	1.98 (0.84)	0.048^S^
3	You appreciated financial compensation if provided after the outbreak	138	92	2.58 (0.79)	2.88 (0.88)	0.294
4	You were unhappy to do overtime	144	96	2.88 (0.72)	2.76 (0.84)	0.246
5	You appreciated special recognition for your job by the Hospital administration	150	100	2.84 (0.74)	2.78 (0.78)	0.163
6	You expected extra financial compensation against your duties during the outbreak	78	52	1.78 (0.64)	1.98 (0.72)	0.021^S^
7	You tried curtailing your contact with the COVID-19 patient (e.g., shorten your trips to isolation wards/rooms)	52	34.7	1.04 (0.73)	1.64 (0.84)	0.034^S^
8	You thought of quitting your job	42	28	1.02 (0.58)	1.42 (0.62)	0.042^S^
9	You felt that employees not directly exposed to COVID-19 avoided you	67	44.7	1.18 (0.68)	1.68 (0.44)	0.614
10	You noticed that employees outside the COVID unit were avoiding patients	102	68	2.08 (0.74)	2.48 (0.58)	0.042^S^
11	If optional, you would have chosen to work in a unit where you would not be exposed to COVID-19	38	25.3	1.04 (0.48)	1.28 (0.84)	0.168
12	You would quit your job if the COVID-19 outbreak recurred	46	30.7	1.08 (0.66)	1.34 (0.58)	0.261
13	You felt angry that your workload increased when compared to employees working non-COVID duties.	28	18.7	0.84 (0.84)	1.02 (0.84)	0.563
14	You thought of calling in sick	22	14.7	0.42 (0.24)	0.78 (0.68)	0.325
15	You called in sick at least once	16	10.7	0.22 (0.38)	0.38 (0.48)	0.412

The responses for the various stress-causing factors showed the impact of the factor in terms of the mean. The main stressors were related to doctors’ own safety as well as the safety of friends and family and fear of having COVID-19 if they developed any respiratory symptoms. Maximum impact is also associated with these stressors. Whereas blaming from higher authorities, colleagues displaying COVID-19-like symptoms, and seeing patients with COVID-19 dying were also very distressing. The scenario is more or less the same for paramedical staff but the intensity of impact for these stressors was found of dissimilar level. The main stressor for them was the apprehension of contracting the infection and colleagues displaying COVID-19-like symptoms. The conflict between your duty and your own safety was an additional major stressor in paramedical. As compared to doctors, paramedical staffs were more stressed with frequent protocol changes, emotional exhaustion, salary conflicts with ongoing duties, every fresh exposure with COVID-positive patients, physical stress, and discomfort with personal protective equipment (PPE) (p-value<0.05) (Table 3).

**Table 3:** Factors that caused stress among staff during the COVID-19 outbreak * Based on Mann-Whitney U Test ^s ^Statistically significant SD: standard deviation

No.	Factors causing stress among healthcare workers	Positive answer (%) (N=150)	Level of stress, Mean (SD)		p-value*
			Doctors	Paramedical	
1	Frequent modification of infection control procedure/protocols	88	2.57 (0.63)	2.78 (0.73)	0.016^S^
2	You could transmit COVID-19 infection to your family or friends	94.7	2.83 (0.81)	2.84 (0.68)	0.652
3	Small mistake or lapse in attention during patient care could infect you or others	96	2.82 (0.58)	2.86 (0.66)	0.218
4	Taking care of your own colleagues sick from COVID-19	69.3	2.07 (0.83)	2.18 (0.68)	0.652
5	Seeing patients with COVID-19 dying in front of you	92	2.72 (0.72)	2.77 (0.76)	0.062
6	Not knowing when the COVID-19 outbreak will be under control	78.7	2.57 (0.68)	2.77 (0.63)	0.014^S^
7	Every time you were exposed to a new COVID-19 suspect patient turns to positive	73.3	2.04 (0.82)	2.27 (0.74)	0.036^S^
8	Lack of specific treatment for COVID-19	81.3	2.42 (0.73)	2.57 (0.64)	0.364
9	News of increase of cases of COVID-19 reported in TV/newspaper	70.7	2.12 (0.43)	2.24 (0.64)	0.542
10	You were emotionally exhausted	68	2.02 (0.54)	2.47 (0.73)	0.026^S^
11	You had physical stress/fatigue	84	2.47 (0.64)	2.78 (0.83)	0.031^S^
12	Colleagues displaying COVID-19-like symptoms	93.3	2.78 (0.42)	2.87 (0.86)	0.149
13	You developed respiratory symptoms and feared that you had COVID-19	94.7	2.87 (0.48)	2.92 (0.68)	0.361
14	You could get COVID-19 infection in the hospital	76	2.12 (0.42)	2.44 (0.89)	0.128
15	Conflict between your duty and your own safety	62.7	1.87 (0.73)	2.17 (0.38)	0.038^S^
16	Seeing your colleagues stressed or anxious	68	2.17 (0.49)	2.47 (0.83)	0.034^S^
17	Blaming from higher authorities	89.3	2.84 (0.63)	2.87 (0.56)	0.062
18	You felt there were some lacunae in protective measures (including enough negative pressure rooms)	84	2.32 (0.32)	2.54 (0.58)	0.248
19	Protective gear cause physical discomfort	70.7	2.12 (0.83)	2.58 (0.73)	0.027^S^
20	Shortage of staff at times for COVID care	74.7	2.18 (0.63)	2.27 (0.88)	0.452

Good quality protective equipment provided by the hospital has the biggest impact in reducing staff stress among healthcare staff. Moreover, items like “none of the staff getting COVID-19 after starting strict protective measures, positive attitude from colleagues in your department, and confidence in the hospital staff in case you got sick from COVID-19” had calmed the doctors. Moreover, the decrease in COVID-19 cases reported in the news and provision of psychiatric services were the additional ease factors to reduces stress among paramedical staff. Psychiatric counselling services were included in the very early stage of the hospital’s action. Most of the healthcare staff agreed that psychiatric services were significantly helping them manage their stress. Factors like seeing colleagues getting better, hoping for extra financial compensation, or provision of immunity boosters by the hospital were reported as stress busters for paramedical more as compared to doctors with statistically significant difference (p<0.05) (Table 4).

**Table 4:** Effective measures to reduce stress * Based on Mann-Whitney U Test ^s ^Statistically significant SD: standard deviation

No	Factors reducing stress among healthcare workers	Positive answer (%)	Mean (SD)		p-value*
			Doctors	Paramedical	
1	Positive attitude and respect from colleagues in your department	96	2.74 (0.64)	2.64 (0.78)	0.562
2	None of the staff getting COVID 19 after starting strict protective measures	97.3	2.78 (0.63)	2.34 (0.42)	0.125
3	Hospital providing regular education programme	93.3	2.64 (0.84)	2.58 (0.66)	0.354
4	Your colleagues who were infected getting better	78.7	2.22 (0.54)	2.58 (0.77)	0.012^S^
5	Good quality Protective equipment provided to you by Hospital	97.3	2.84 (0.44)	2.72 (0.62)	0.082
6	Hospital enforcing stringent infection control procedure	90.7	2.42 (0.38)	2.58 (0.56)	0.562
7	Providing regular Psychiatric services	90.7	2.68 (0.38)	2.62 (0.74)	0.084
8	Decrease in COVID-19 cases reported in the news	62.7	1.75 (0.54)	2.63 (0.57)	0.028
9	The likelihood that you would get extra compensation for your exposure to COVID-19	49.3	1.14 (0.73)	1.63 (0.81)	0.042^S^
10	All healthcare professionals working together on the frontline	58.7	1.64 (0.44)	1.97 (0.71)	0.367
11	Confidence in the hospital staff in case you got sick from COVID-19	85.3	2.72 (0.78)	2.59 (0.69)	0.362
12	Sufficient rest or time off	82.7	2.34 (0.68)	2.24 (0.89)	0.340
13	Sharing jokes or humor among colleagues	64	1.84 (0.24)	1.98 (0.77)	0.286
14	Hospital providing nutriments/immunity boosters	52	1.41 (0.69)	1.83 (0.74)	0.034^S^

Personal coping strategies are always essentially coexisting with stress. Most of the healthcare staff responded positively to items like “followed strict personal protective measures (e.g., mask, gown, handwashing, etc.), kept separate clothes for work/used disposable scrubs provided by the hospital, and considered every patient admitted to the hospital as having COVID-19 infection and using full protective gear even if the patient was COVID-19 negative and avoided going out in public places to minimize exposure from COVID-19” were the highest approved coping strategies adopted to assuage stress. On the assessment of the impact of various coping strategies, the strategies like "kept separate clothes for work/used disposable scrubs provided by the hospital" among doctors and "followed strict personal protective measures (e.g. mask, gown, handwashing, etc.)" are found most workable and "vented emotions by crying, screaming, etc" the least effective coping strategy among doctors and paramedical staff as well. For doctors, strategies like separating clothes used at the workplace and considering each patient as COVID positive irrespective of COVID status were more effective for personal coping as compared to the paramedical staff. Additionally, for the paramedical staff, avoiding public outing, getting help from physicians, getting busy with household chores, and avoiding overtime to reduce exposure were more effective for personal coping as compared to doctors (p<0.05) (Table 5).

**Table 5:** Personal Coping strategies used by the staff to alleviate stress * Based on Mann-Whitney U test ^s ^Statistically significant

No.	Personal coping strategy used by healthcare workers	Positive answer (%)	Mean (SD)		p-value*
			Doctors	Paramedical	
1	Followed strict personal protective protocols/measures (e.g. Mask, gown, hand washing, etc.)	100	2.82 (0.63)	2.78 (0.57)	0.256
2	Kept separate clothes for work/used disposable scrubs provided by Hospital to minimize transmission	100	2.92 (0.43)	2.84 (0.68)	0.028^S^
3	Considered every patient admitted to the hospital as having COVID 19infection and using full protective gear even if the patient was COVID 19 negative	100	2.82 (0.53)	2.62 (0.42)	0.012^S^
4	Update about COVID-19, its symptom, mechanism of transmission & prevention, etc.	96	2.73 (0.93)	2.79 (0.33)	0.086
5	Avoided going out in public places to minimize exposure from COVID-19	100	2.62 (0.47)	2.82 (0.69)	0.016^S^
6	Did relaxation activities, e.g., involved in prayers, sports, exercise, etc.	85.3	2.51 (0.85)	2.78 (0.52)	0.042^S^
7	Chatted with family and friends to relieve stress and obtain emotional support	88	2.63 (0.53)	2.72 (0.49)	0.062
8	Talking to yourself and motivating to face the COVID-19 outbreak with a positive attitude	45.3	1.02 (0.71)	1.31 (0.47)	0.042^S^
9	Got help from family physicians or hospital psychiatrist to reduce your stress and get reassurance	28	0.68 (0.40)	1.82 (0.32)	0.018^ S^
10	Tried to be busy at home in activities that would keep your mind away from COVID-19	26.7	0.48 (0.72)	1.12 (0.41)	0.042^S^
11	Avoided doing overtime to reduce exposure to COVID-19 patients in hospital	25.3	0.42 (0.53)	1.28 (0.35)	0.036^S^
12	Avoided media news about COVID-19 and related fatalities	65.3	1.62 (0.98)	1.96 (0.33)	0.564
13	Vented emotions by crying, screaming, etc.	10.7	0.22 (0.63)	0.52 (0.78)	0.268

We tried to explore motivators for health care workers to continue working during any like situation or other infectious disease outbreak. Recognition from management and supervisors for the extra efforts and supply of similar adequate personal protective equipment by the hospital are the most effective motivational factors among doctors, whereas among motivating factors that could make the paramedical staff more willing to join the team in the future, the most crucial was having recognition from management and supervisors for the extra efforts and available cure or vaccine for the disease followed by disability benefits if disabled from the disease. Not forced to do overtime among paramedical staff and reduced working hours during outbreaks among doctors were found to be the least favourable motivators.

Motivational factors like family compensation in case of death at the workplace, financial recognition of efforts, disability benefits in case of disease-related disability development, reducing working hours were more effective for paramedical staff in case of future outbreaks as compared to doctors (p<0.05) (Table 6).

**Table 6:** Motivational factors for future outbreaks * Based on Mann-Whitney U test ^s ^Statistically significant SD: standard deviation

S.No	Motivational factors for future outbreaks	Mean (SD)		p-value*
		Doctors	Paramedical	
1	Similar adequate efficient personal protective equipment supply by the Hospital	2.87 (0.67)	2.48 (0.51)	0.254
2	Available cure or vaccine for the disease	2.86 (0.76)	2.78 (0.58)	0612
3	Family support	2.68 (0.41)	2.74 (0.81)	0.245
4	Compensation to family if disease related death at work	2.38 (0.73)	2.56 (0.53)	0.035^S^
5	Financial recognition of efforts	2.52 (0.37)	2.68 (0.47)	0.022^S^
6	Disability benefits if disease related disability	2.42 (0.71)	2.77 (0.55)	0.038^S^
7	Recognition from management and supervisors for the extra efforts and duties during outbreak	2.88 (0.31)	2.79 (0.48)	0.648
8	Psychiatric help and therapy made available in work place to help reduce stress and anxiety	2.28 (0.44)	2.18 (0.39)	0.218
9	Not forced to do overtime	1.44 (0.47)	1.29 (0.84)	0.482
10	Reduced working hours during outbreaks	1.38 (0.41)	1.68 (0.63)	0.036^S^

## Discussion

Healthcare workers indeed play a crucial role in current pandemic management and control. This makes them susceptible to anxiety, stress, and fear of acquiring the infection [10,11]. Previous outbreaks and epidemics, such as SARS-CoV-1, H1N1 influenza, and the Ebola virus, have been shown to have major short- and long-term psychological impacts on frontline healthcare staff [12,13]. In the early stages of the COVID-19 pandemic, infected HCWs accounted for 29% of all COVID-19 patients hospitalised [14]. Furthermore, the stress experienced by HCWs will have an impact on their performance in terms of attention, cognitive functioning, and clinical decision-making [15]. Despite the institutions' strict adherence to infection prevention and control measures as per recommendations for COVID-19, HCWs are still vulnerable to higher levels of anxiety. The latest emerging existence of a virus with uncertain contagiousness, rapidity of spread, and degree of knowledge associated with it [16] is similar to that observed during the H1N1 Influenza pandemic [17].

Currently, India faces the most critical phase of the pandemic and our HCWs across the country are facing a situation like never before. However, every outbreak or epidemic differs with respect to its distribution, pathogenesis, transmissibility, infectivity, and fatality. Since no two disease outbreaks are similar, they leave their own unique impact on the healthcare staff handling that scenario [18]. 

Nurses and medical workers encountered emotional turmoil during the COVID-19 outbreak, which was consistent with previous research in SARS-affected hospitals [19]. Our staff's anxiety and nervousness were normal in every outbreak, with varying degrees of severity. According to surveys in the United Kingdom, 28% of doctors and 32% of nurses scored above the "emotional distress" threshold in General Health Questionnaire (GHQ)-12 [20]. 

The avoidance of overtime and expectation of extra compensation during an outbreak with special recognition were strongly favoured by our staff and were observed in other epidemics as well [9,21]. However, the most important finding was that most of the workers felt that continuing to work is secondary to their ethical and professional obligation towards their profession, which was a finding consistent with the view that HCWs consider it unethical to desert their professional liabilities and accountabilities in order to protect themselves or their families [4,22].

This research reflects the desired attempt to set up certain provisions to meet the psychological needs of front-line health care staff in similar circumstances by investigating the secret. The results of this study were close to those of Mitchell et al. who discovered extreme stress among nursing staff while battling a vancomycin-resistant enterococci (VRE) outbreak [23]. Therefore, it is possible that such psychological reactions to extreme stress may be common among nurses caring for victims during a highly infectious epidemic. As a result, it's worth wondering whether such psychological responses to intense stress are normal among nurses caring for victims during a highly contagious epidemic. Among the numerous stressors associated with the COVID-19 outbreak, the staff's primary concern was safety. At our organisation, healthcare staff expressed serious concerns about contracting COVID-19 and then transmitting it to others; this finding is expected and reproduced from previous similar studies [11].

Some other stressors for them were seeing their colleagues display COVID-19-like symptoms, patients dying in front of them from COVID-19, and blame from higher authorities. These factors were seen among HCWs who faced SARS, but with lesser intensity [9].

The hospital staff in our sample was also very worried about the epidemic's unknown period and the lack of care for the disease. Concerns regarding infecting family members [24], the virulence of the disease and inappropriate facilities, feelings of confusion, insufficient staffing level, nosocomial spread, and personal danger are among the notified stressors, which are in accordance with previous findings of others [25]. Frontline healthcare workers, such as those employed in ICUs and respiratory units, were at high risk of infection [26], and it has been reported that they were afraid of contracting the virus and spreading it to their families, mates, or coworkers [27].

During infectious epidemics, HCWs have also been shown to undergo severe stress. High levels of anxiety were normal in such outbreaks, according to studies of the psychological effect of SARS on hospital workers [28]. As a result, hospitals should often emphasize implementing different stress-reduction techniques and providing psychological support to their workers. Our staff appreciated the influence of strict infection management practice guidelines and the provision of appropriate personal protective equipment in reducing their tension, as shown by their responses. There were two key factors that were found to be effective in easing the tension. Positive attitudes from colleagues and the fact that nosocomial transmission of COVID-19 was absolutely stopped after following the prescribed strict precautions were important factors for the healthcare staff. During the COVID-19 crisis, our staff continued to operate in an atmosphere of hope and assurance of personal safety, and these two factors can be crucial in maintaining staff during an epidemic. 

Our employees' personal coping mechanisms offer further insight into the COVID-19 epidemic. Their extreme alertness to the situation is reflected in their use of stringent protective measures for all patients, the use of disposable scrubs at work, and the use of semiquarantine to minimise outside exposure. Dealing with every disease necessitates this level of vigilance and caution. 

Psychosocial treatments in a hospital's Emergency Department helped physicians and nurses select more adaptive coping mechanisms, according to Phua, Tang, and Tham [29]. 

When asked what factors would motivate workers to continue working through potential epidemics, factors such as protection, disease awareness, special pay, and recognition after the outbreak came up. Some of these factors reflect what has been reported in other epidemics [29]. Khalid et al. concluded in their study that HCWs’ ethical allegiance to their duty, coupled with strict control measures, recovery from infectivity amongst colleagues, and recognition for their efforts helped them navigate through the pandemic and would motivate them to perform optimally in future outbreaks [4].

Limitations of the study

This cross-sectional observational study was conducted with a small sample size within a limited duration, and thus, could not address the long-term psychological effects on healthcare workers. Only persons with a known psychological disorder were excluded. Existence of additional psychological stressor due to routine life were not considered. 

*Future Scope of the Study*: This study could be executed as longitudinal research with a control group and extended to multiple centres to validate the findings of the current study.

## Conclusions

It is extremely important to note, as learned from this study, that disease outbreaks/pandemics are always a stressful condition for health care professionals and resulted in some psychological trauma. All the HCWs in our study believed that service provision is their ethical duty in pandemic and appreciated any financial benefit. Fear of transmitting the infection to family members, seeing dying COVID-19 patients or colleagues displaying COVID-19 symptoms has been a stressor for more than 90% of HCWs. Additionally, positive attitude reception by colleagues, good PPE kits provision, strict adherence to infection control protocol, and provision of psychiatric services were the factors that helped reduce stress among HCWs. Hence, it is necessary that these secondarily traumatised team members should be always observed, educated, and properly handled. Certain personal coping strategies adopted by health care staff should be well addressed and motivated if scientifically sustainable.

The findings of our study helped in surfacing the key areas of concern and expectancy of the healthcare workers from the system. This can further act as a guiding principle for policy formulation in terms of psychiatric assistance, stress monitoring, and unbiased feedback mechanism. While stressing sanitation, temperature control, and fever management, we suggest that the Department of Health and Family Welfare provide psychiatric preparedness and stress monitoring for healthcare teams in their outbreak response preparation.
